# Surgical techniques for aortic valve xenotransplantation

**DOI:** 10.1186/s13019-021-01743-0

**Published:** 2021-12-28

**Authors:** Jennie H. Kwon, Morgan Hill, Brielle Gerry, Steven W. Kubalak, Muhammad Mohiuddin, Minoo N. Kavarana, T. Konrad Rajab

**Affiliations:** 1 Division of Cardiothoracic Surgery, Department of Surgery, Medical University of South Carolina, Charleston, USA; 2 Department of Regenerative Medicine and Cell Biology, Medical University of South Carolina, Charleston, SC USA; 3 Xenotransplantation Program, University of Maryland School of Medicine, Washington, DC, USA

**Keywords:** Xenotransplantation, Aortic valve transplantation, Aortic valve replacement, Partial heart transplantation

## Abstract

**Background:**

Heart valve replacement in neonates and infants is one of the remaining unsolved problems in cardiac surgery because conventional valve prostheses do not grow with the children. Similarly, heart valve replacement in children and young adults with contraindications to anticoagulation remains an unsolved problem because mechanical valves are thrombogenic and bioprosthetic valves are prone to early degeneration. Therefore, there is an urgent clinical need for growing heart valve replacements that are durable without the need for anticoagulation.

**Methods:**

A human cadaver model was used to develop surgical techniques for aortic valve xenotransplantation.

**Results:**

Aortic valve xenotransplantation is technically feasible. Subcoronary implantation of the valve avoids the need for a root replacement.

**Conclusion:**

Aortic valve xenotransplantation is promising because the development of GTKO.hCD46.hTBM transgenic pigs has brought xenotransplantation within clinical reach.

## Introduction

Valvular heart disease affects approximately 2.5% of the U.S population and causes over 25,000 deaths each year [1, 2]. Severe cases are typically treated using heart valve replacement [3]. However, conventional heart valve prostheses are marred with drawbacks. Mechanical valves are durable but highly thrombogenic. Therefore they require permanent anticoagulation. Anticoagulation for mechanical valves is associated with a risk of major bleeding or thromboembolic events of 1% per patient-year [4–7]. Anticoagulation also poses particular challenges for female patients during their reproductive years and for patients with active lifestyles [8–10]. Therefore, the American College of Cardiology and the American Heart Association recommend bioprosthetic valve prostheses in patients of any age for whom anticoagulant therapy is contraindicated [11]. Bioprosthetic valves do not require anticoagulation but are prone to early structural valve degeneration. This puts young and middle-aged patients at a high risk for re-interventions to replace the degenerated bioprosthesis [4, 12]. The same is true for homografts. Additionally, neither chemically fixed bioprosthetic valves nor mechanical valves have the potential to grow with children. Therefore, children with conventional valve replacements are committed to serial re-operations for successively larger implant exchanges. These patients often endure up to 5 or more open heart operations in their lifetime [13]. Therefore, there is an urgent clinical need for heart valve replacements that are durable without the need for anticoagulation and that grow adaptively with children. For the last fifty years, approaches to deliver such valve replacements have focused on tissue engineering. However, all tissue engineered valves have failed in clinical translation [14–18]. This is a critical barrier to progress.

**Fig. 1:**
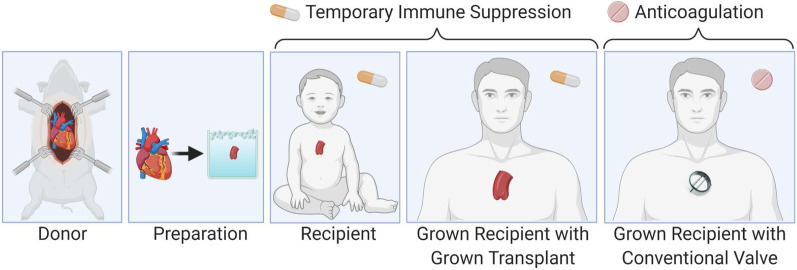
Heart valve xenotransplantation involves temporary immune suppression until the transplanted valve can be exchanged for an adult-sized prosthetic valve in the grown child

**Fig. 2:**
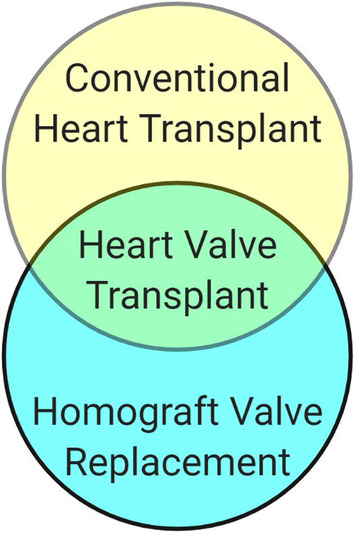
Heart valve transplantation overlaps with conventional heart transplantation from a immunology perspective and homograft valve replacement from a surgical perspective

We propose heart valve xenotransplantation as a new approach to deliver growing heart valve replacements that are durable without the need for anticoagulation (Fig. 1). Unlike conventional porcine or bovine bioprostheses, xenotransplants contain live cells that allow the graft to avoid thrombogenesis, heal from mechanical damage to valvular extracellular matrix, and grow with children. This comes at the cost of immunosuppression. Heart valve xenotransplantation resembles homograft valve replacement from a surgical perspective and cardiac xenotransplantation from a transplant immunology perspective (Fig. 2). This approach is promising because the development of GTKO.hCD46.hTBM transgenic pigs has brought xenotransplantation is within clinical reach [19, 20]. Here we describe surgical techniques for aortic valve xenotransplantation that were developed in a human cadaver model.

## Donor operation

**Fig. 3:**
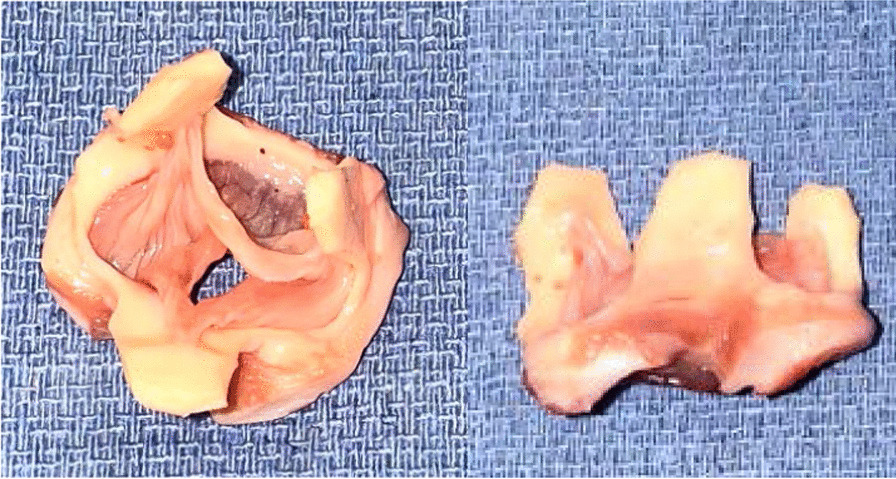
The donor valve is prepared by trimming the ventricular muscle and mitral valve to within 2–3 mm of the aortic valve annulus. The sinuses of valsalva were also trimmed, leaving only the commissural posts suspending the aortic valve behind. Panel A shows the superior view, panel B shows the lateral view of the valve

Porcine donor hearts (Animal Technologies, Tyler, TX) are excised and preserved in ice-cold University of Wisconsin solution (Global Transplant Solutions, Spartanburg, SC) in typical fashion for a standard human donor cardiectomy. To prepare the donor aortic valve, the donor aorta is divided just distal to the sinotubular junction. The aortic root is then dissected to the aortic annulus. The anterior leaflet of the mitral valve and the ventricular muscle are carefully trimmed to within 2–3 mm of the aortic valve annulus. The sinuses of valsalva are also trimmed, leaving only the commissural posts suspending the aortic valve within (Fig. 3). The donor aortic valve is kept in ice-cold University of Wisconsin solution until ready for transplantation.

## Recipient operation

**Fig. 4:**
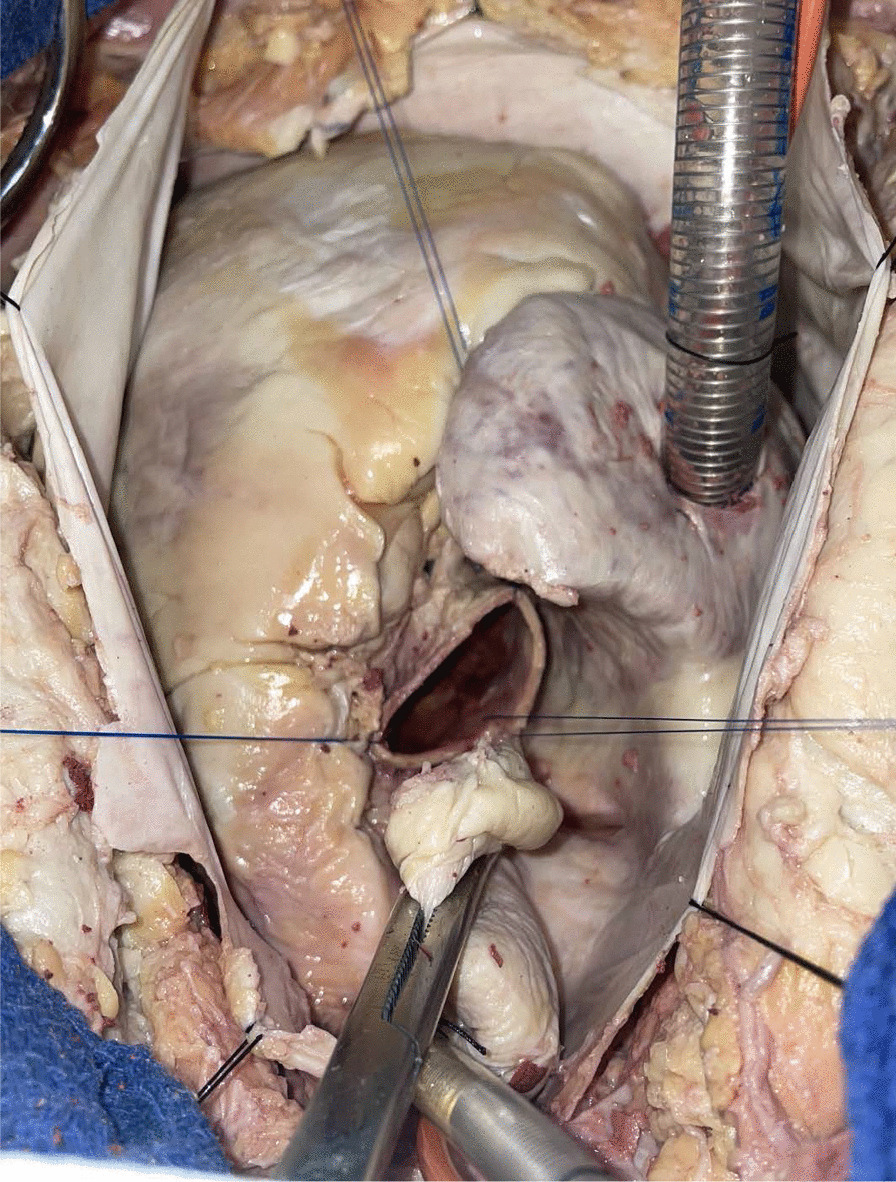
The human recipient aorta is opened and the diseased aortic valve excised similar to a conventional aortic valve replacement. Transection of the aorta above the sinotubular junction optimizes the exposure

**Fig. 5:**
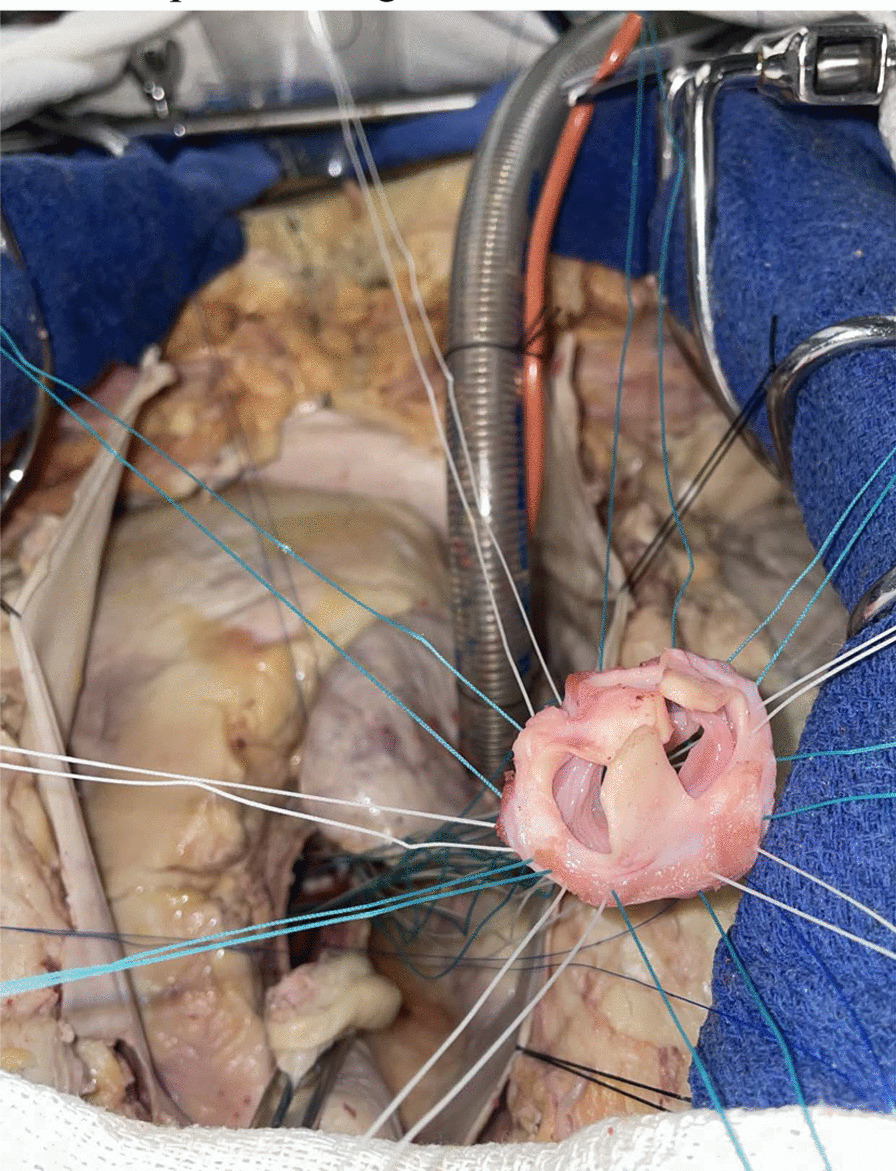
Mattress sutures are placed through the native aortic annulus and the donor valve annulus to allow parachuting the donor valve into the recipient aortic root

**Fig. 6:**
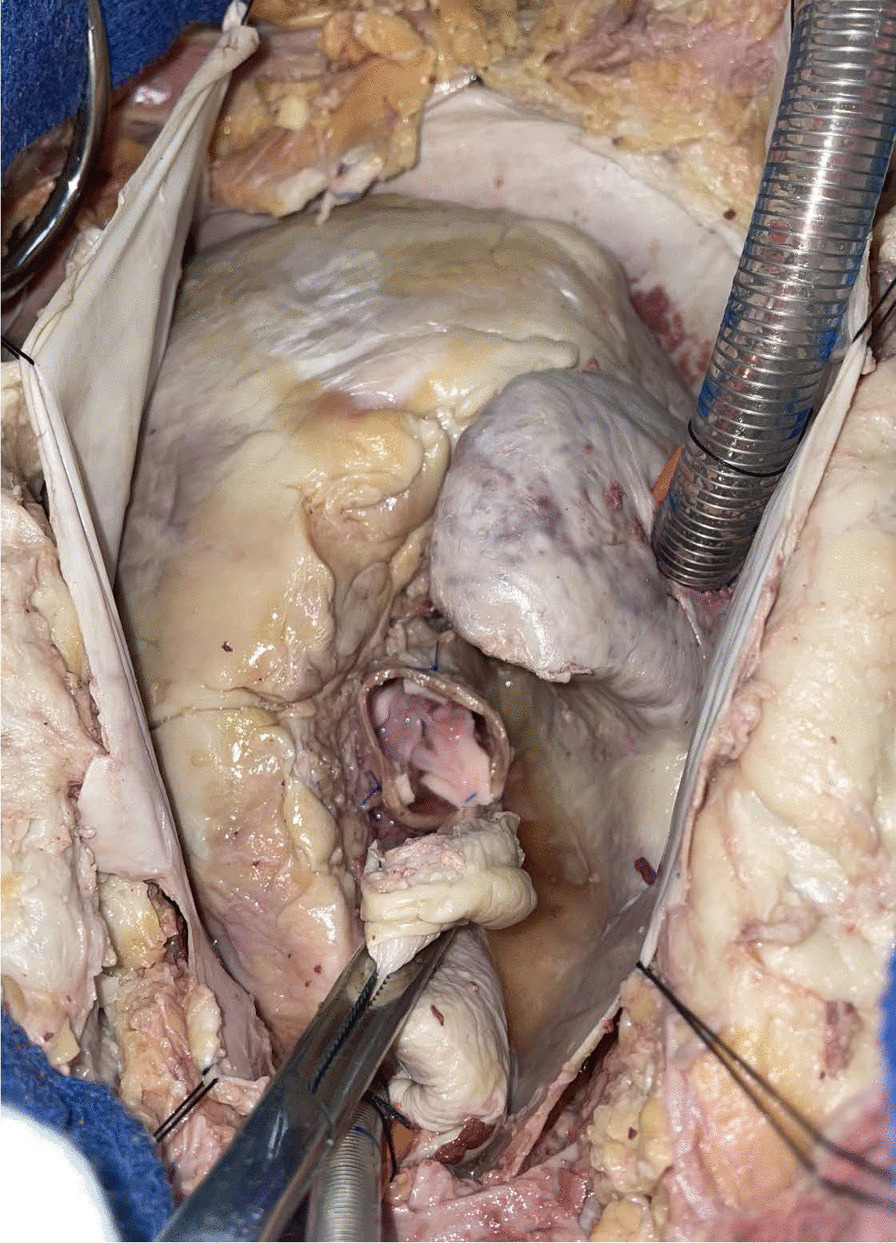
The donor valve commissural pillars are suspended in the recipient aorta using mattress sutures

Human cadavers donated to the Medical University of South Carolina were used to develop the recipient operation. The heart and aorta are exposed by midline sternotomy. Aortic and right atrial cannulae for cardiopulmonary bypass and a right superior pulmonary vein vent are inserted in the usual fashion. The ascending aorta is cross-clamped and antegrade cardioplegia is delivered into the root. The aorta is divided just distal to the sinotubular junction and the aortic valve is exposed by placing retraction sutures at the level of each commissure. The diseased aortic valve is excised (Fig. 4). Pledgeted horizontal mattress sutures are placed in the annulus of the recipient aorta using 4-0 braided polyester suture. The sutures are then placed through the donor aortic valve annulus (Fig. 5). The donor aortic valve is oriented 120 degrees counterclockwise relative to the recipient aortic valve so that the donor right coronary sinus is aligned with the recipient left coronary sinus. This moves the muscle rim under the right coronary sinus of the graft posteriorly, where it is less likely to protrude into the left ventricular outflow tract. The donor valve is then parachuted into the aortic root and each suture is tied. The donor valve commissural pillars are suspended in the recipient aorta with 4-0 prolene sutures (Fig. 6). The ascending aortotomy is closed and the operation is completed in the usual fashion.

## Discussion

Partial heart xenotransplantation is a new approach to deliver growing heart valve replacements that are durable without the need for anticoagulation. Initially the entire heart is recovered from the donor animal and transported on ice within 6 h to the recipient. The valve is then removed from the donor heart on the backtable and immediately transplanted into the recipient. Here we describe surgical techniques for valve implantation developed in a human cadaver model. Several factors were considered during the development of these techniques.

### Root replacement versus subcoronary valve implantation

Heart valve transplantation can be accomplished by root replacement or subcoronary valve implantation. The aortic root replacement technique is technically easier, because the valve and root are implanted as a functional unit. Therefore matching of the graft size to the host annulus is more forgiving and function of the valve is less prone to surgical error [21]. In contrast, the subcoronary implantation technique has a steeper learning curve [21, 22]. This technique was initially described by Barratt-Boyes for homografts and was subsequently adapted as an alternative to the root replacement technique in the Ross procedure [23, 24]. For heart valve xenotransplantation, the subcoronary technique has the advantage of minimizing the donor tissue burden on the host.

Controversy exists over the use of the subcoronary vs root replacement technique with regard to their long-term durability and outcomes [25]. While some studies cite a lower reoperation rate and incidence of aortic regurgitation after root replacement compared to subcoronary implantation, others found root replacement to be associated with an increased risk of perioperative death [25–27]. However, when early reoperations for aortic regurgitation due to plain technical failure was excluded, no major differences between the root replacement and subcoronary implantation technique were found [21].

### Modifications to the subcoronary technique

McGriffin and Kirkland found that failure of grafts implanted by the subcoronary technique is often due to geometric distortion of the donor valve commissural pillars, which must be aligned just as they were in the donor [28]. However, this alignment may not always be possible due to distortion of the recipient aortic root. Modification of the subcoronary technique with retention of the noncoronary sinus maintains the geometric relationship of the two commissures on each side of the sinus [28]. While this modification has the disadvantage that the additional tissue increases the donor tissue burden, it should be kept in mind depending on the anatomy of the recipient aortic root. For example, aneurysmal dilation of the non coronary aortic sinus could be an indication for retaining the noncoronary sinus of the donor graft.

It is also possible to invert the donor valve into the left ventricular outflow tract to complete the annular suture with a continuous suture line [28]. However, while this maneuver saves cross-clamp time, it can be difficult in a small aortic root [29].

### Rotation of the donor valve

The porcine aortic valve differs from the human aortic valve because a larger part of the annulus is supported by ventricular muscle [30]. Therefore, the donor valve was rotated by 120 degrees counterclockwise, bringing the donor non-coronary sinus against the recipient left coronary sinus. This maneuver moves the proximal muscle rim of the donor valve posteriorly an minimizes its protrusion into the left ventricular outflow tract [29]. Barrett-Boyes described a 180 rotation to avoid sewing muscle to muscle and so that the more difficult anterior sutures, which bite into muscular tissue of the patient’s septum, pass through the fibrous aortic ring of the graft [23]. Notably, rotation of the graft cannot be performed if the noncoronary sinus is retained.

### Size mismatch

A possible obstacle is size mismatch of the donor graft and host aortic root. Ideally, this is addressed by selecting appropriately sized donor valves with a defined diameter. The ideal donor aortic valve has an internal diameter of 2–3 mm less than the recipient annular diameter [29, 31]. Alternative techniques to address size mismatch include the use of everting versus non-everting annular sutures and root enlargement or root plication techniques.

In case of a small host aortic valve that goes beyond mere size mismatch, there are several alternative technical options for xenograft valve transplantation. Firstly, a posterior root enlargement (Manugian or Nicks) can be performed. Secondly, an anterior root enlargement (Konno) can be performed. An anterior root enlargement can increase the root size by a greater degree, but is technically more complex. Thirdly, the host root can be replaced with the xenograft root. The xenograft root can also be harvested with additional tissue for a combined aortic root transplantation and Konno root enlargement. This will provide the greatest degree of root enlargement.

### Valve competency

After valve transplantation, competency may be tested with saline injection into the root prior to completion of the aortotomy closure and removal of the aortic cross clamp. While this technique may identify large perivalvular leaks or severe malcoaptation of the valve leaflets, this is an insensitive test for the transplanted valve. Recently, a device has been designed to evaluate aortic valve repair under physiologic pressures, which may be used to test transplanted valves in the future [32].

### Transplant immunobiology and developmental biology

Investigation into the immunobiology and developmental biology of transplanted valves is ongoing, but graft tolerance and growth are favorably supported by experience with conventional heart transplantation. For example, valvular dysfunction is not clinically recognized even in patients dying of fulminant myocardial rejection [33]. The incidence of aortic valve intervention after heart transplantation is exceedingly low, occurring in 0 in 867 and 2 in 1466 consecutive heart transplant recipients in two large institutional series at a median follow-up greater than 4 years [34, 35]. In addition, our group has demonstrated that structural integrity, cellular viability, and cellular proliferation in neonatal rat aortic grafts is maintained even after 48 h of cold storage in University of Wisconsin preservation solution [36]. This suggests that wide geographic transport of donor valves is feasible without adversely affecting graft survival.

### Comparison with the Ross operation

The Ross operation is currently the most widely used valve replacement strategy to provide a growing, durable, and thromboresistant valve in children and younger adults. This strategy has several drawbacks which will make valve transplantation favorable. In children, reintervention on the right ventricle to pulmonary artery conduit poses a significant morbidity and mortality risk for these growing patients [37]. Neonates and infants have particularly dismal outcomes after Ross operation with a 24–30% in-hospital mortality [38, 39]. Finally, for patients with dysfunctional pulmonary valves such as those with truncus arteriosus, the Ross operation is not a viable option.

Regarding growth, Ross pulmonary autografts and aortic annuluses after conventional heart transplantation follow appropriate somatic growth curves following excision and implantation [40, 41]. However, the Ross valve is a pulmonary valve that physiologically operates under much lower pressure. Therefore there is concern for inappropriate dilation of the root and early failure of the valve. On the other hand, the retained growth potential and developmental biology of transplanted valves has yet to be specifically explored. So far, valve growth after xenotransplantation has not been examined in vivo.

## Conclusion

Conventional heart valve prostheses do not contain live cells. Therefore these prostheses fulfil the structural functions of native heart valves, but they cannot fulfil their biological functions, namely adaptive growth, avoiding thrombogenesis, and self-repair. This severely limits the lifespan of the conventional heart valve prostheses in growing children and young adults with contraindications for anticoagulation [42, 43]. While glutaraldehyde fixation decreases the antigenicity of bioprosthetic heart valves to avoid immune rejection, it also may underlie several of the pathophysiological features of early structural valve degeneration. For example, the loss of glycosaminoglycans and elastin as well as collagen cross-linking resulting from glutaraldehyde treatment makes bioprosthetic valves rigid and adversely affects their mechanical properties [12]. Glutaraldehyde fixation may also contribute to prosthesis-related dystrophic calcification, as it has been found that treatment of porcine aortic valves by glutaraldehyde results in their gradual calcification accompanied by a depletion of calcium ions from the culture medium [44]. Bioprosthetic valves are also more prone to degeneration because of the altered structure of the chemically treated extracellular matrix [12]. Again, the absence of live cells in these valves mean that mechanical damage to the extracellular matrix is not repaired.

Heart valve xenotransplantation is a new approach to deliver growing heart valve replacements that are durable without the need for anticoagulation. This addresses an urgent clinical need for growing heart valve replacements for children, as well as young adults with contraindications for anticoagulation. Therefore heart valve xenotransplantation can spare these patient populations from serial re-interventions for heart valve exchanges. The transplanted valves are fresh and contain live cells that allow the valve to perform biological functions such as growth, avoiding thrombogenesis, and self-repair of the extracellular matrix. These advantages do not come without disadvantages. Most importantly, patients who receive transplanted valves require immunosuppression until the transplanted valve can be replaced for a conventional prosthesis in the grown child or older adult.

**Fig. 7:**
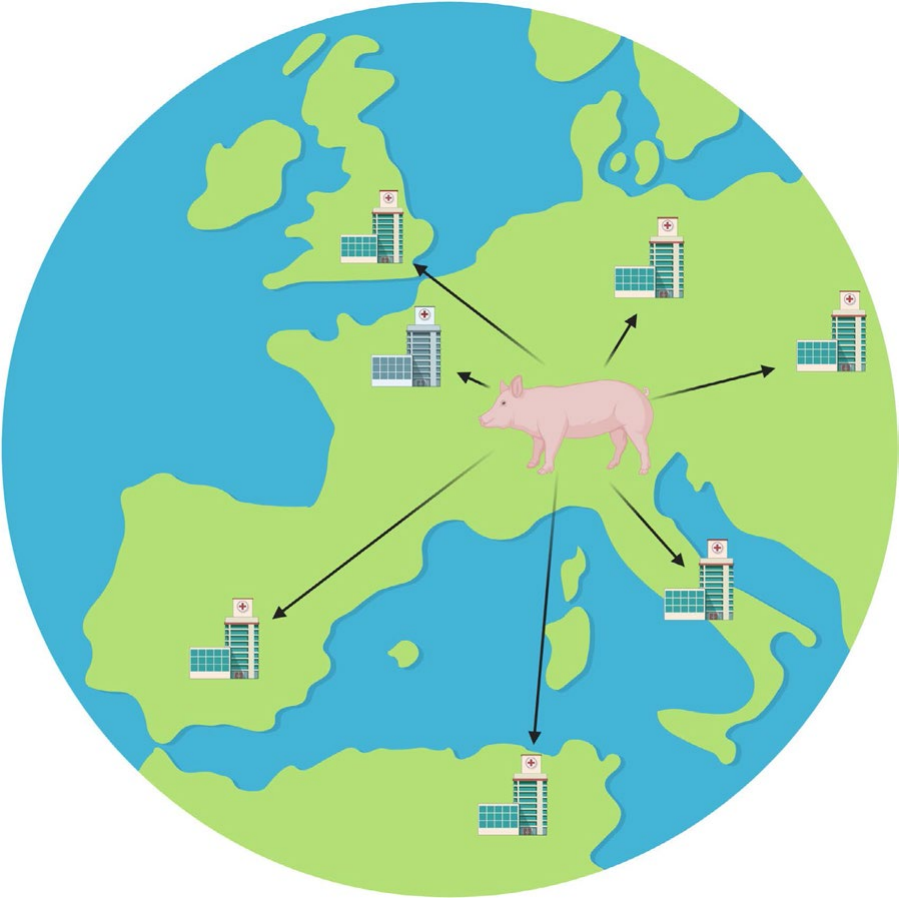
Heart valve xenotransplantation raises the possibility that appropriate sized donor valves will be delivered from regional facilities to hospitals “just in time” for transplantation

In summary, heart valve transplantation is a new operation to deliver heart valve replacements with the ability to grow, self-repair, and avoid thrombogenesis. Xenotransplantation is a promising approach because it would allow for donor valves of appropriate size to be delivered to hospitals “just-in-time” for transplantation (Fig. 7).

## Data Availability

Not applicable.

## Abbreviation

GTKO.hCD46.hTBM: Alpha 1–3 galactosyltransferase gene knockout pigs, which express human complement regulatory protein CD46 and human thrombomodulin
